# Supplementary material for: Characterization and Pathogenicity of Flavobacterium psychrophilum Isolated from Rainbow Trout (Oncorhynchus mykiss) in Korea
Source: Microorganisms. 2023 Oct 12;11(10):2546. doi: 10.3390/microorganisms11102546 (PMC10609541; doi:10.3390/microorganisms11102546)
Supplement: Supplementary file 1 [file microorganisms-11-02546-s001.zip › microorganisms-2640523-Table S1.pdf]

Supplementary Table S1. Enzymes activities produced by *F.psychrophilum* and comparison among the other strains.

Enzyme	Isolates of Finland(N=37) (Madetoja et al, 2001)	Isolates of Sweden(N=8) (Madetoja et al, 2001)	Isolates of Estonia(N=3) (Madetoja et al, 2001)	Isolates of Turkey(N=20) (Kum et al, 2008)	Isolates of Canada(N=42) (Hesami et al, 2010)	Isolates of Danish (N=15) (Christiansen et al, 2016)	FPRT1
Alkaline phosphatase	+	+	+	+	+	+	+
Esterase (C4)	+	+	+	-	-	+	+
Esterase lipase (C8)	+	+	+	+	+	+	+
Lipase (C14)	+	+	+	-	-	-	-
Leucine arylamidase	+	+	+	+	+	+	+
Valine arylamidase	+	+	+	+	+	+	+
Cystine arylamidase	variable	+	+	-	+	-	-

Trypsin	variable	+	+	-	+	-	-
a-Chymotrypsin	-	-	-	-	-	-	-
Acid phosphatase	+	+	+	+	+	+	+
Naphtol-AS-BI-phospho-hydrolase	+	+	+	+	+	+	+
α-Galactosidase	-	-	-	-	-	-	-
β-Galactosidase	-	-	-	-	-	-	-
β-glucuronidase	-	-	-	-	-	-	-
α-Glucosidase	-	-	-	-	-	-	-
β-Glucosidase	-	-	-	-	-	-	-
N-Acetyl-β-glucosaminidase	-	-	-	-	-	-	-
α-mannosidase	-	-	-	-	-	-	-

α –fucosidase	-	-	-	-	-	-	-

In this result means += positive, - = negative.
