# Supplementary figures and images for: Characterization and Pathogenicity of Flavobacterium psychrophilum Isolated from Rainbow Trout (Oncorhynchus mykiss) in Korea
Source: Microorganisms. 2023 Oct 12;11(10):2546. doi: 10.3390/microorganisms11102546 (PMC10609541; doi:10.3390/microorganisms11102546)

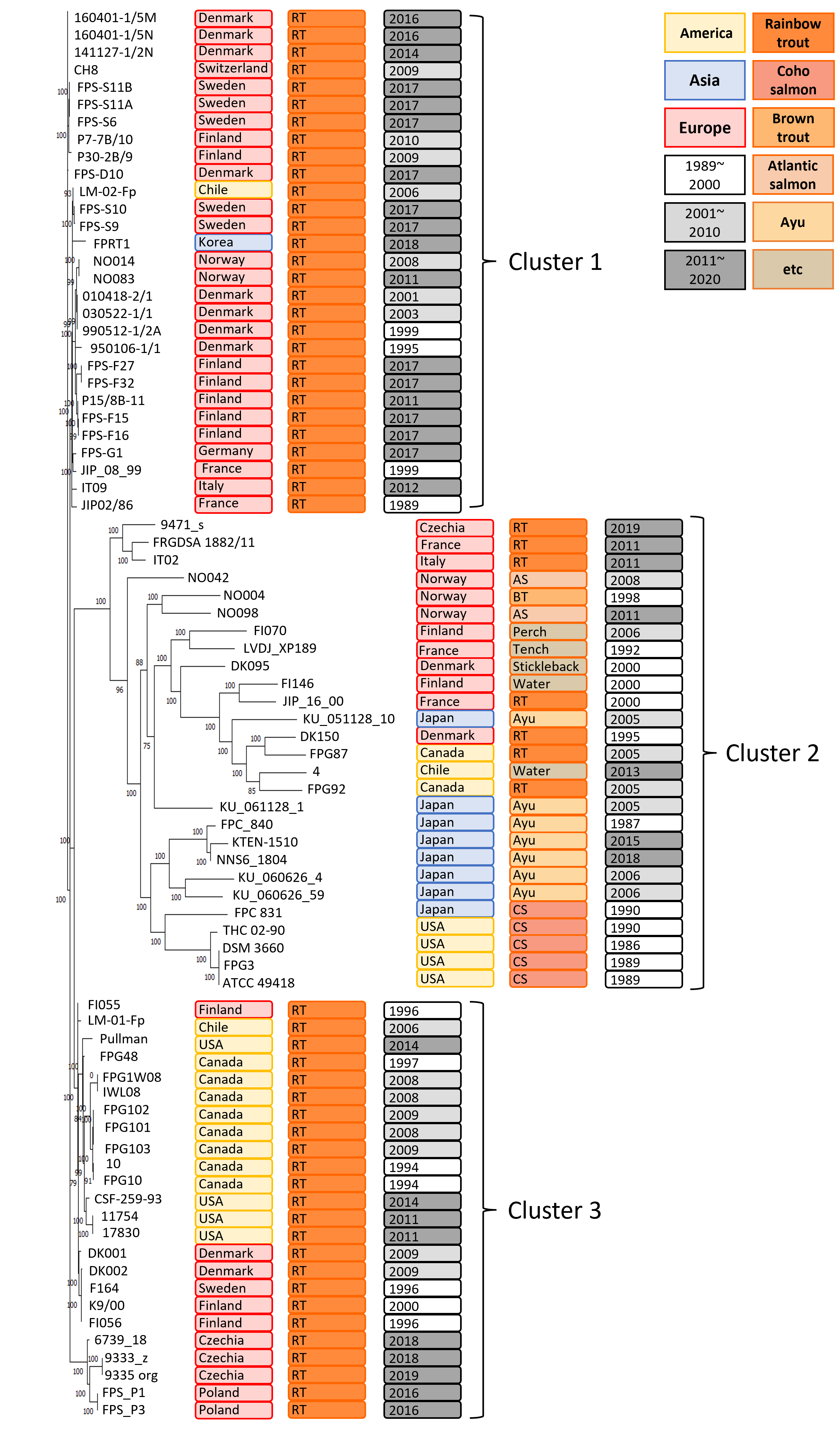

Supplement: Supplementary file 1 [file microorganisms-11-02546-s001.zip › Supplementary Figure S1.tif]
